# Terahertz *in vivo* imaging of human skin: Toward detection of abnormal skin pathologies

**DOI:** 10.1063/5.0190573

**Published:** 2024-03-11

**Authors:** X. Qi, K. Bertling, J. Torniainen, F. Kong, T. Gillespie, C. Primiero, M. S. Stark, P. Dean, D. Indjin, L. H. Li, E. H. Linfield, A. G. Davies, M. Brünig, T. Mills, C. Rosendahl, H. P. Soyer, A. D. Rakić

**Affiliations:** 1 School of Electrical Engineering and Computer Science, The University of Queensland, Brisbane QLD 4072, Australia; 2 Dermatology Research Centre, Frazer Institute, The University of Queensland, Woolloongabba QLD 4102, Australia; 3 School of Electronic and Electrical Engineering, University of Leeds, Leeds LS2 9JT, United Kingdom; 4 OscillaDx Pty Ltd, Brisbane, Queensland, Australia; 5 General Practice Clinical Unit, Faculty of Medicinee, The University of Queensland, Herston QLD 4029, Australia

## Abstract

Terahertz (THz) imaging has long held promise for skin cancer detection but has been hampered by the lack of practical technological implementation. In this article, we introduce a technique for discriminating several skin pathologies using a coherent THz confocal system based on a THz quantum cascade laser. High resolution *in vivo* THz images (with diffraction limited to the order of 100 *μ*m) of several different lesion types were acquired and compared against one another using the amplitude and phase values. Our system successfully separated pathologies using a combination of phase and amplitude information and their respective surface textures. The large scan field (50 × 40 mm) of the system allows macroscopic visualization of several skin lesions in a single frame. Utilizing THz imaging for dermatological assessment of skin lesions offers substantial additional diagnostic value for clinicians. THz images contain information complementary to the information contained in the conventional digital images.

## I. INTRODUCTION

As the largest organ in the human body, the skin plays a vital role in protecting against microorganisms, dehydration, ultraviolet light, and mechanical damage. Abnormal skin conditions are a widespread health problem affecting people of all ages, which include seborrheic keratosis, angioma, neurofibroma, actinic keratosis, mycosis fungoides, and skin cancer. Skin cancer is the most common type of cancer in Australia and the USA. Each year, more than 18 000 Australians are diagnosed with invasive melanoma while over a million new cases of non-melanoma skin cancers (i.e., basal and squamous cell carcinomas: the two most common types of skin cancer worldwide) are treated.^1,2^ The current diagnostic skin imaging techniques, which include digital photography,^3–5^ dermoscopy,^6–8^ and reflectance confocal microscopy,^9–11^ are based either on assessing the lesion morphology and color or on assessing the morphology of the lesion at the cellular level (histopatholog).^12^ These methods tend to rely on microscopic or macroscopic inspection for identification of the disease, and suspicious lesions will typically be biopsied, but the sensitivity of histopathologic examination of difficult cases even in expert hands is still only approximately 80%.^13^ A recent investigation shows that diagnoses spanning moderately dysplastic naevi to early stage invasive melanoma were neither reproducible nor accurate.^14^ Use of new standardized classification tools that are employing improved diagnostic technology might reduce the potential for miscommunication and management errors. There is an unmet need for a system capable of objectively determining how far cancerous lesions have progressed, in order to achieve optimal balance between early detection and overdiagnosis of melanoma.^2^

Over the past two decades, terahertz (THz) sensing has been heralded as a unique biomedical imaging technology.^15–17^ THz science and research may provide foundational understanding of biomaterials and could underpin valuable new technologies to improve human healthy.^18^

However, the translation of these promises into a clinically viable technology has been hampered by the lack of an appropriate platform suitable for rapid acquisition of high resolution THz images. Due to the non-ionizing photon energy, high sensitivity to water content in living tissues,^19–22^ and potential to detect molecular fingerprints,^23–25^ THz imaging is a safe and sensitive modality suitable for biomedical *in vivo* imaging. THz *in vivo* imaging is eminently suitable for skin imaging due to limited penetration depth owing to the high absorption of THz waves in living tissues.^26–29^ THz imaging and spectroscopy are highly sensitive to the structure of skin tissue molecules, which can provide functional information associated with the molecular structure of biomarkers that are beyond morphology. There is a significant body of the published work demonstrating the relationship between THz contrast and malignancy in skin.^30,31^ In this study, we offer a radically different use of THz technology to aid dermatological assessment of skin lesions: We combine high resolution amplitude and phase images obtained by a coherent THz receiver with surface texture analysis to deliver a new capability to aid dermatological assessment and to discriminate different pathologies aided by computer analysis of THz data. The quantum cascade laser (QCL)-based THz imaging system uses laser feedback interferometry (LFI)—the laser itself acting as the source and the detector.^32,33^ Over the past decade, this approach has established itself as one of the most promising sensing technologies due to high-sensitivity,^34,35^ coherent detection, self-alignment, and high frame-rate.^36–38^ This technology constitutes a major leap beyond the current commercially available THz imaging systems and provides a clear pathway toward commercially viable, low-maintenance THz sensing, and imaging systems, operating in the genuine THz spectrum with acceptable imaging times.

In this study, we conducted THz *in vivo* imaging study of human skin lesions, including angioma, seborrheic keratosis, benign naevus, neurofibroma, basal cell carcinoma (BCC), mycosis fungoides (also known as cutaneous T-cell lymphoma or CTCL), actinic keratosis, photodamaged skin, tattoo, and scars (a total of 29 skin pathologies from 14 participants) with a novel THz QCL confocal imager at 2.85 THz. As defined by the FDA, “A first in human study is a type of study in which a device for a specific indication is evaluated for the first time in human subjects,” which is referred to as first-in-man. Traditional feasibility studies are the preferred path for design changes incorporating next-generation technologies. The large scan area combine with the high resolution and small minimum resolvable feature (diffraction limited) of the system allows of hundreds of thousands individual pixel measurements across the healthy skin and hundreds to thousands of individual measurements across even quite small (millimeter scale) pathologies. This allowed the acquired images to exhibit clear contrast between normal skin and the range of pathologies, which are directly observable from the THz amplitude and phase images and not in the visible light photographs. Coherent images of several different lesion types were compared against one another using the amplitude and phase values. Our system successfully separated different pathologies using a combination of phase and amplitude information and their respective surface textures due to the large number of pixels interrogated across the individual lesions. This proof-of-concept first-in-man study outlines the path forward for improving the system design and future clinical studies for all QCL-based THz imaging systems.

## II. RESULTS

### A. THz *in vivo* images of different types of skin lesions

In this *in vivo* study, we measured 29 skin lesions from 14 human participants with a novel THz confocal imager using a THz QCL. The different skin pathologies covered in this study include angioma, seborrheic keratosis, several benign naevi, neurofibroma, basal cell carcinoma (BCC), mycosis fungoides, actinic keratosis, photodamaged skin, a tattoo, and scars. The comprehensive list of lesion types per participant as well as the corresponding anatomical locations can be found in Table I.

**TABLE I.** List of Participants and details of areas scanned for the *in vivo* study.

ID	Sex	Age	Location	Diagnosis
1	Male	68	Back (lower)	Angioma
			Back (upper)	Angioma
				Naevus
				Seborrheic keratosis
2	Male	41	Right hand	Naevus
			Back (upper)	Naevus
			Back (lower)	Naevus
			Abdomen	Naevus
3	Male	68	Left arm (upper)	Angioma
				Naevus
				Seborrheic keratosis
				Scar
				Photodamage
			Back (upper)	Seborrheic keratosis
				Photodamage
4	Male	64	Back (upper)	Naevus
				Photodamage
			Back (lower)	Scar
5	Male	74	Back (upper)	Angioma
			Back (upper)	Angioma
				Seborrheic keratosis
				Scar
			Back (lower)	Angioma
				Neurofibroma
6	Female	60	Back (upper)	Naevus
7	Male	53	Left arm (upper)	Naevus
8	Male	81	Left hand	Actinic keratosis
			Left arm	Actinic keratosis
			Back (upper)	BCC
			Back (lower)	BCC
9	Male	30	Right arm (upper)	Naevus
10	Female	28	Back (upper)	Naevus
11	Male	20	Back (lower)	Scar
12	Male	54	Left wrist	CTCL (mycosis fungoides)
			Left forearm	CTCL (mycosis fungoides)
			Right hand	CTCL (mycosis fungoides)
13	Female	42	Left arm	Naevus
			Back (lower)	Naevus
				Tattoo
			Back (lower)	Tattoo
14	Female	41	Right arm	Scar

**FIG. 1.**
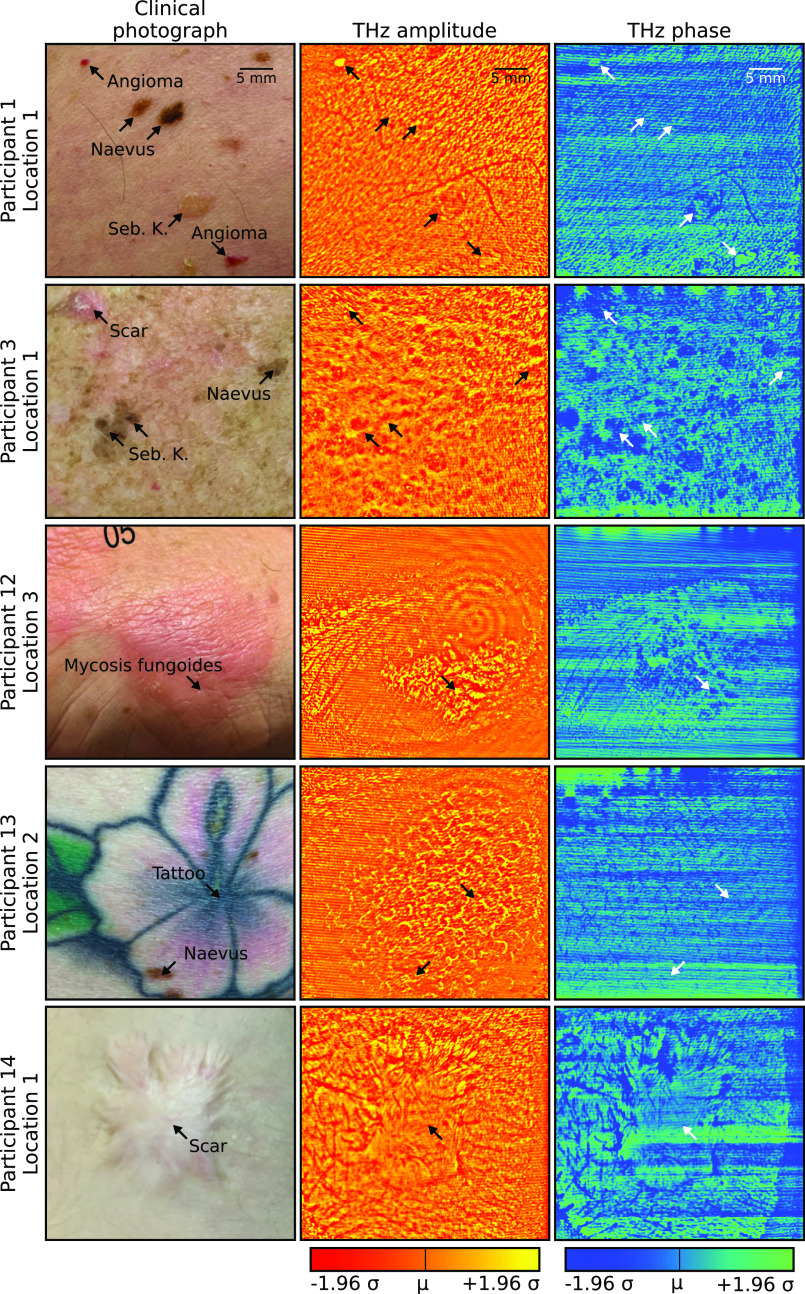
Overview of different skin lesion types included in the study. The amplitude and phase images were scaled to ±1.96 standard deviations around the mean value of the respective images.

Figure 1 comprises of the digital photo, THz amplitude, and THz phase images for clinically typical skin lesions covered in this study. The THz confocal imager produced high-resolution amplitude-phase image pairs as well as their respective surface textures. The importance of surface textures for the discrimination between different pathologies will be addressed later (see Sec. IV). Clinical photographs of the corresponding skin regions (Fig. 1) offer complementary information than that in the THz images.

Both Figs. 1(a) and 1(b) show several types of skin pathologies in the same scanning area of 50 × 40 mm^2^. The images in Fig. 1(a) show two angiomas, two flat naevi, and a seborrheic keratosis. Strong contrasts were observed for both angiomas and the seborrheic keratosis when compared to the surrounding normal skin in the THz amplitude and phase images, as indicated by the arrows in the images. The contrast of the two naevi in the THz images is not as evident as in the visible photo, which might be owing to no notable fingerprint of the melanin content at the emission frequency from the QCL at 2.85 THz.^31^ The images in Fig. 1(b) show a participant with photodamaged skin on his arms and back, which can be seen from the skin texture in both the visible and THz images for a skin lesion located on the upper arm. These images [Fig. 1(b)] show a scar, a seborrheic keratosis, and a benign nevus, which are clear in both the visible and THz images. Figure 1(c) shows images from a participant who has mycosis fungoides mainly on his arms and back, where the triangular shape of the mycosis fungoides can be clearly observed in the THz images. The tattoo in Fig. 1(d) is not evident in the THz images as the ink is typically deposited at depths of 1–2 mm, which is beyond the penetration depth of the THz field. The scar shown in Fig. 1(e) formed from a boiling water burn when the participant was 10-year-old without proper treatment. The scar can be clearly observed from both THz amplitude and phase images, including the irregular skin margin of the burnt area. The contrast observed is most likely from the structural collagen of the scar along with differing water content compared with the normal healthy skin.

**FIG. 2.**
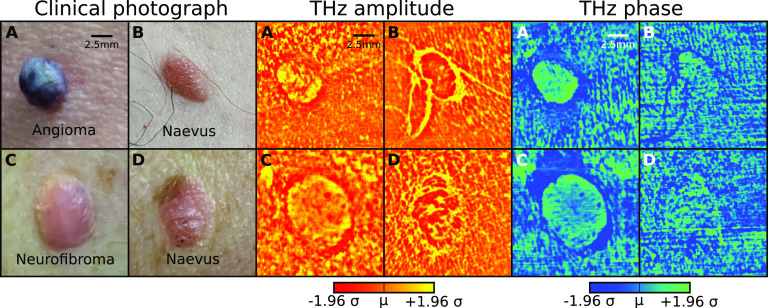
A close-up of large skin lesions with multiple complementary features in amplitude and phase images. The amplitude and phase images were scaled to ±1.96 standard deviations around the mean value of the respective images.

There are several benefits of using the narrow linewidth source (like a THz QCL) with high emission power (∼mW) as used in our confocal imager. The spatial resolution of our *in vivo* images is at the diffraction limit at 2.85 THz (∼110 *μ*m). This spatial resolution is higher than conventional THz imaging technology based on broadband sources (∼350 *μ*m from TDS systems.^19,39,40^) More interestingly, in some lesions [Figs. 2(a)–2(d)], the THz contrast in the amplitude and phase images actually reveal subtle features (both structures and textures in both healthy skin and lesions) not visible in the clinical reference photographs. The spectral information of the THz images is not always encoded in just one domain but alternates between the amplitude and phase information as well as the surface texture (Fig. 2, for example). Notably, these texture metrics can only be reliably obtained from high resolution THz images, as presented here, therefore, ruling out the current commercial THz technologies.

### B. Skin lesion discrimination

**FIG. 3.**
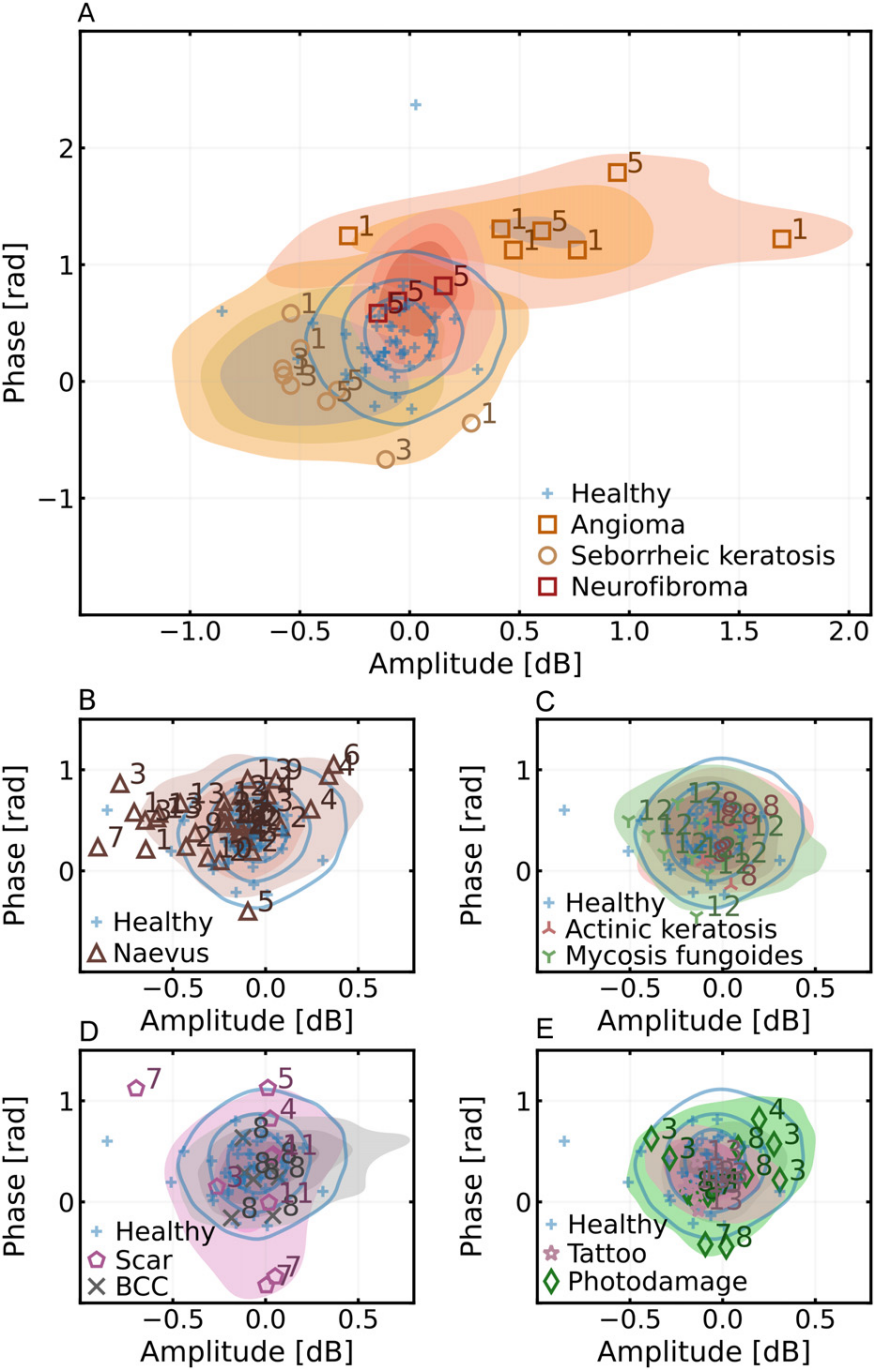
Distribution of amplitude-phase values of different skin lesions represented as kernel density estimates. Shaded regions correspond to the joint distributions estimated over all participants and all regions-of-interest. The markers represent the mass center of each individual region interest with a number corresponding to the participant.

**FIG. 4.**
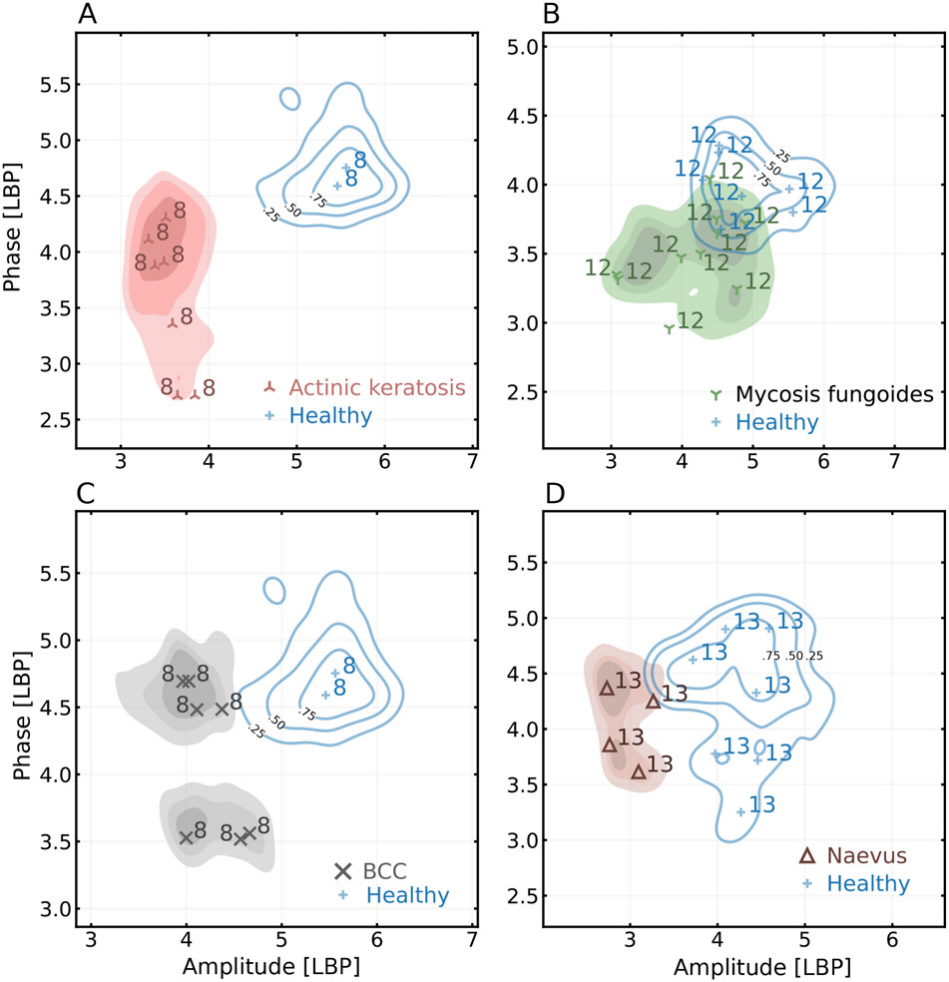
Distribution of amplitude and phase local binary pattern textures of different skin lesions represented as kernel density estimates. Shaded regions correspond to the joint distributions estimated over all participants and all regions of interest. The markers represent the mass center of each individual region interest with a number corresponding to the participant.

This study imaged various *in vivo* skin lesions with a novel confocal THz QCL imager based on laser feedback interferometry. The resulting images suggest that most lesions will stand out from healthy skin in the amplitude and phase images representations and can be visually observed, as shown in Figs. 1 and 2. Furthermore, we investigated the possibility of augmenting the clinical classification of lesions by comparing the bivariate amplitude-phase distributions of the THz images. The ability to delineate between different skin lesions was investigated both in the amplitude-phase domain (Fig. 3) as well as with surface texture analysis [amplitude and phase local binary pattern analysis (for details, see Sec. IV)] (Fig. 4).

The amplitude-phase distribution of each lesion type was approximated by computing the kernel density estimate of the distribution by combining similarly sized regions-of-interest (ROI) across all participants. In the amplitude-phase domain, angioma, seborrheic keratosis, and neurofibromas exhibited joint distributions substantially different healthy skin [Fig. 3(a)]. The contrast difference for these lesions could possibly be attributed to difference in geometry, since all three have the physical appearance of a raised papule.^41^ This is unlikely to be entirely correct since other lesion types (e.g., scars and dermal naevi) also exhibit raised profiles without similar effect in contrast. More likely, the difference can be explained by differences in composition, which then translate to varying levels of THz absorption and penetration depth. It is feasible that these three lesions can be automatically detected based on the amplitude-phase values alone. There was significant overlap and no notable difference in the amplitude-phase distributions between healthy skin, photodamaged skin, actinic keratosis, CTCL, and tattoos [Figs. 3(c) and 3(e)]. The distributions of naevi, scars, and BCC also predominantly overlapped with healthy skin [Figs. 3(b) and 3(d)] but with substantially varied shapes of distributions.

While the amplitude-phase distributions of various lesions did overlap, they also exhibited distinct geometric patterns under visual observation. For instance, the physical appearance of CTCL was clearly distinguishable from a healthy skin in the THz images (Fig. 1). The bivariate distributions were, thus, recalculated by substituting the phase-amplitude values with corresponding lesion textures approximated with local binary patterns (Fig. 4). This additional analysis step revealed that lesions of actinic keratosis [Fig. 4(a)], CTCL [Fig. 4(b)], BCC [Fig. 4(c)], and dermal nevus [Fig. 4(d)] were indeed different from corresponding healthy skin when investigated in the texture domain.

The remaining lesions (benign naevi, tattoo, scar, and photodamage) could not be differentiated from healthy skin [Figs. 3(b) and 3(E)]. For the tattoo, the ink is typically deposited at depths of 1–2 mm, which is beyond the penetration depth of the THz radiation frequency range utilized in the study. The ability to filter out tattoos is probably beneficial as they are seldom of clinical relevance but might obscure actual features of interest in typical dermoscopy.^42^ While scars exhibited similar amplitude-phase values as the healthy skin, they often possess distinct edges which can be identified via edge detection. The ability of THz imaging to discern dermal naevi but not the other two nevus types could potentially be due to dermal naevi having closer proximity to skin surface.

Textures obtained through local binary pattern analysis indicated good intra-participant differentiation between healthy skin and actinic keratosis, CTCL, BCC, and dermal nevus (Fig. 4). In some instances, the imaged lesions could also be identified solely by their boundary morphology [e.g., the nevus in Fig. 2(b) or the scar in Fig. 1] despite there being major overlapped distributions in the amplitude or phase when compared to healthy skin.

## III. DISCUSSION AND CONCLUSION

The results of this first-in-man clinical imaging study are in line with our earlier *ex vivo* experiments with fixed sectioned skin samples,^31^ despite the differences in imaging orientation (i.e., longitudinal vs cross-sectional). Notably the presence of water *in vivo* does not necessarily hinder the separation of lesion types compared to dehydrated *ex vivo* samples. The results also reaffirm earlier reports of THz spectroscopy, exhibiting contrast between nevus types^26^ and BCC.^30^ Finally, in comparison to a recent review article in skin cancer THz imaging,^43^ the confocal imaging technique reported herein provides the best current combination of spatial resolution and image acquisition speed in single frequency (narrow band) THz imaging.

Despite the relatively high quality of THz images, some minor instrumentation artifacts still persist in the system. These consist of imaging artifact caused by the residual mechanical vibration of the system and poor material choice of the imaging window. In this study, these artifacts were corrected through image processing but in the future could be fully eliminated through more optimized hardware design. Furthermore, the single frequency design of the current system could in the future be augmented with a multi-laser system encompassing a wider frequency range. Inclusion of additional frequencies is likely to yield complementary information and make the technique more akin to hyperspectral imaging in the THz domain.

In this study, the analysis of the THz images was limited to identifying potential features for delineating between pathological and healthy skin, due to the low number of samples compared to the number of different lesion types. Likewise, the collected images lacked the histopathological references measurements meaning that accuracy of the sample labels was dependent only on the expert clinical evaluation. The latter limitation stems from the fact that all participants were predominantly healthy volunteers and, thus, unlikely to undergo excision of the lesions imaged in the study. Despite these limitations, the automatic classification of skin lesions with THz images seems evidently feasible, provided that higher number of samples and histopathological reference measures are collected. The current study serves a proof of concept for utilizing THz imaging for detecting skin pathologies under *in vivo* conditions.

The technique was capable of differentiating between different lesion types based on varying morphology and THz absorption. The observed morphology was also unique in the sense that it contained contrast information from THz range, which is not necessarily present in the visible spectrum.

The clinical relevance of the complementary information in THz images, however, still remains to be established. Confocal THz QCL imaging could have a role in improving diagnosis of skin lesions in dermatology.

## IV. METHODS

### A. Experimental setup

**FIG. 5.**
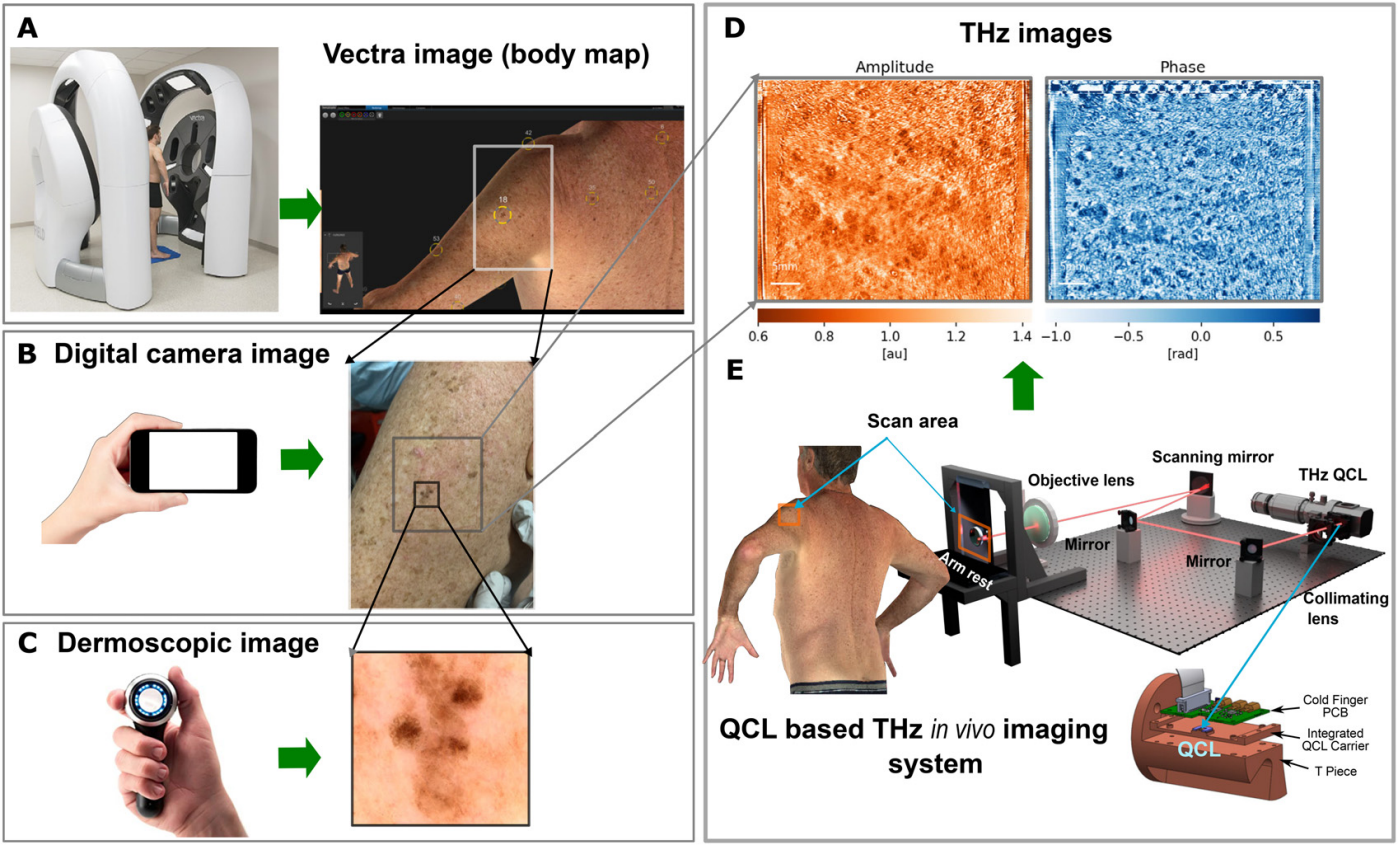
THz *in vivo* images of skin lesions and alongside images from conventional noninvasive imaging equipment including: (a) The 3D image (body map) acquired using a VECTRA WB360 from Canfield Scientific; (b) The digital camera image from a conventional digital camera; (c) The dermoscopic image using a conventional handheld dermatoscope; (d) The THz amplitude and phase images from the QCL based THz *in vivo* imaging system (e), where the laser beam from the THz QCL is collimated by a collimating lens and then guided by two mirrors onto a scanning mirror and then focused by an objective lens and incident upon a skin lesion.

This study utilized QCL-based THz *in vivo* LFI imaging alongside conventional noninvasive imaging equipment: VECTRA WB360 whole body 3D imaging system (WB360 Imaging System, Canfield Scientific, Parsippany, NJ, US), a digital camera, and a dermatoscope (Fig. 5). The VECTRA system imaged the entire skin surface of the participant with macro resolution in a single capture. The proprietary software of the VECTRA system enabled the selection of skin lesions most suitable for THz imaging in terms of body location [Fig. 5(a)]. Areas around the upper limbs and the dorsum were preferred due to flat surfaces being easier to image with the current THz imaging setup. The clinician then acquired dermoscopic images with a handheld device to provide a clinical classification for each of the selected the skin lesion [Fig. 5(c)]. The lesions selected in the previous step were scanned with the THz imaging system around an area [50 mm (horizontal) × 40 mm (vertical) 0.4 megapixels] [Fig. 5(d)]. Finally, the scanned skin lesions (with a trace left on the skin to indicate the exact scanning area) were once more photographed with a conventional digital camera [Fig. 5(b)].

The LFI QCL confocal THz imaging system [Fig. 5(e)] was recently used by our group to image *ex vivo* skin samples.^31^ In brief, the laser beam from a THz QCL was collimated by a plastic lens [*f* = 30 mm, Tsurupica TM (Terahertz Super lens) Broadband, Inc.] and then guided by a set of 75 mm flat mirrors onto a mechanical 50 mm scanning mirror (Optics In Motion LLC) before being focused by an objective lens (*f* = 50 mm, Tsurupica, Broadband, Inc.) and incident upon *in vivo* human skin. In this typical LFI system, the laser emission from the THz QCL reflects back into the laser cavity from each pixel of the skin sample during scanning process, mixes with the intra-cavity electric field, and generates a measurable self-mixing (SM) signal in terminal voltage of the laser at each skin pixel.^32^ The variations in the terminal voltage of the laser, which contains the information about the skin samples, were extracted and amplified as described in Ref. 44. At each pixel of the imaged skin we obtain a time-domain SM signal, which was translated into frequency domain through Fourier transform and provides the spectrum of the interferometric signal, including its amplitude and phase at the frequency of the SM waveform. The amplitude (in dB) and phase (in degree) at each pixel of a skin sample were used to create the THz amplitude and phase images of the sample, respectively. This enables the coherent system to provide both structural and functional information of the skin lesions.

The THz QCL used in the *in vivo* imaging system consisted of a 12 *μ*m-thick GaAs/AlGaAs nine-well phonon-assisted active region with a design frequency of 2.9–3.2 THz as described.^45^ Briefly, the laser was grown by solid-source molecular beam epitaxy on a semi-insulating GaAs substrate, with the active region grown between doped upper 50 nm-thick and lower 700 nm thick GaAs contact layers. Using photolithography and wet chemical etching, the wafer was processed into 150 *μ*m wide surface-plasmon ridge waveguide structure with the substrate thinned to 200 *μ*m. The device was then mechanically cleaved to define a ridge of length 1.8 mm. The THz QCL was driven by a custom-built laser pulse driver and operated at 50 K by a Stirling cryocooler system, as described in Ref. 44. The driving current was set as ramped pulse train (pulse duration: 600 ns, duty cycle: 14.3%) with the amplitude ranging from 1.516 to 1.062 A (454 mA current sweep), which produced the LFI signal by tuning the emission frequency from the laser (∼900 MHz). The operation frequency was 2.85 THz as determined by.^46^ The system parameters are shown in Table II.

**TABLE II.** System parameters for LFI imaging setup.

System parameters	Fast THz–QCL–LFI
Frequency	2.85 THz with 900 MHz freq. sweep
Output power	2 mW peak
Image scan area	50 (H) × 40 (V) mm^2^
Image pixel area	1000 (H) × 400 (V) (0.4 M pixels)
Pixel size	50 (H) × 100 (V) *μ*m
Resolvable feature size	∼100 *μ*m (diffraction limited)
Image acquisition time	150 s (mechanically limited)

### B. Participant selection and imaging procedure

The current study performed *in vivo* THz imaging of skin lesions of volunteer participants (N = 14, four female) ranging from 20 to 81 years in age (average = 51.7 ± 17.9 years). Participants were selected based on having skin lesions on their bodies at body sites suitable for THz imaging, resulting in images from a total of 29 (50 × 40 mm^2^) regions [Fig. 7(c)]. Each imaged area contained at least one lesion and regions representative of healthy skin. In addition to healthy skin, the list of pathologies included: actinic keratosis, angioma, BCC, CTCL, nevus, neurofibroma, scar, seborrheic keratosis, and tattoo (Table I).

The majority of skin lesions were selected and diagnosed by a senior dermatologist (HPS) based on the clinical and dermoscopic images provided by the aforementioned Vectra System. After participant induction (i.e., filling the *Participant Information Consent Form*, discussing the THz imaging procedure, answering any concerns or questions, and obtaining a signed consent), the selected skin area was examined by a dermatologically trained clinician (FK) to identify and photograph suitable lesions. The skin area was then scanned by the THz imaging system. During the scan, the participant was required to rest their arm (or other body site imaged) against a quartz reference window (see Fig. 5) for approximately 150 s for acquiring a 50 × 40 mm^2^ 0.4 M pixel THz image. As part of participant safety, the system was evaluated against the AS/NZS IEC 60825.1:2014 standard (the same as the US ANSI Z136.1:2014 standard and EU Directive 2006/25/EC of the European Parliament and Council). Based on all relevant standards, the maximum exposure by our device (<0.002 J/cm^2^) is two orders of magnitude below the maximum permissible exposure (>0.063 J/cm^2^)). Therefore, the device was determined to be safe to use on human skin. Other studies at similar THz wavelengths also show this level of exposure to be safe.^47,48^

Additionally, an “Adverse Event” log was setup as per the ICH Harmonized Tripartite Guideline for Good Clinical Practice for any adverse events during the THz imaging on the day and having follow up phone calls on days 1, 3, and 7. No adverse effects due to the THz imaging were observed either during or after the imaging process.

### C. Image processing and statistical analysis

The interferograms scanned from each pixel are first processed on the field-programmable gate array (FPGA) of the driver/receiver board. The individual interferogram traces are averaged 32 times followed by trimming the ends of the traces and subtraction of the baseline signal. The traces are then windowed and subsequently fast Fourier transformed (FFT) to extract the amplitude and phase values corresponding to the fundamental frequency component of the SM waveform. These pixels are then collated into columns and rows with known positions and passed to the controlling PC to generate the raw rastered THz amplitude and phase images. The values of the amplitude images were converted from reflectance intensity to insertion loss according to

$$\text{IL}(\text{dB})=-10\;{\text{log}}_{10}I,$$ (1)

where *I* is the intensity of reflected radiation. Phase information was stored in radians.

**FIG. 6.**
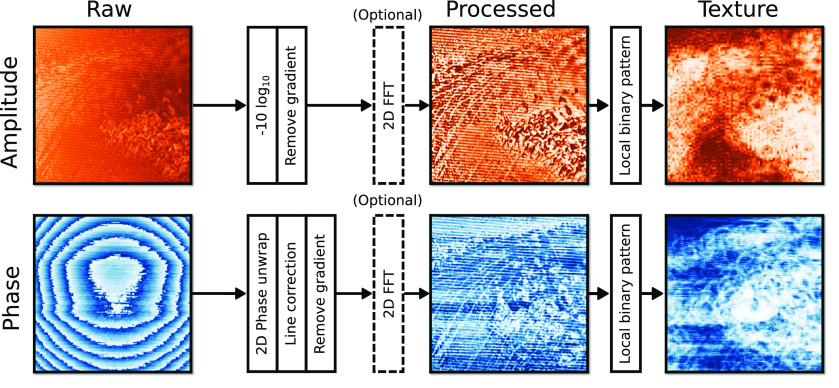
Post-processing pipeline used for the current measurements.

The collected raw images were post-processed in Python 3.8+ using the scikit-image^49^ module. Post-processing was necessary due to relatively high noise content in some of the images, which consisted primarily of motion artifacts and artifacts originating from the high reflectivity of the sample-window interface. Processing steps and parameters of the algorithm were initially fine-tuned to produce high contrast images while eliminating most of the noise contamination. Post-processing of amplitude images (Fig. 6) consisted of evaluation and removal of background variation followed by optional 2D-FFT notch filtering to remove periodic artifacts (only performed for images with notable artifacts) using manually defined stop-bands. In order to retain as much of the original contrast as possible, the histograms of the filtered images were matched to the histograms of the unfiltered images. Phase images were first phase unwrapped and line corrected to remove horizontal fringe artifacts.

Next, the post-processing of mirrored amplitude images, i.e., consisting of background variation elimination and 2D-FFT filtering is done when necessary. In order to quantify the geometric patterns exhibited by different lesions, each processed amplitude-phase image pair was subjected to texture analysis using rotation invariant local binary patterns (LBP).^50^ Amplitude textures were evaluated by first compressing the image by a factor of 0.15 and then computing LBP using a four pixel radius with 12 point angular quantization. For phase textures, the compression factor was set to 0.256, radius to 12 pixels, and angular quantization to 36 points.

**FIG. 7.**
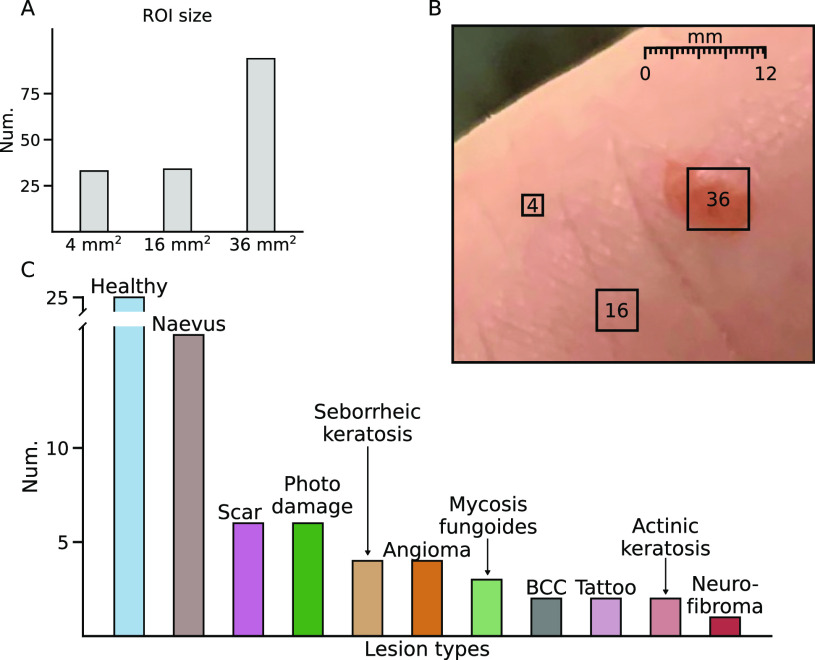
(a) Histogram of different region-of-interest sizes used in the study. (b) Visual demonstration of the relative sizes of the three ROI types. (c) Histogram of different skin lesions included in the study.

**FIG. 8.**
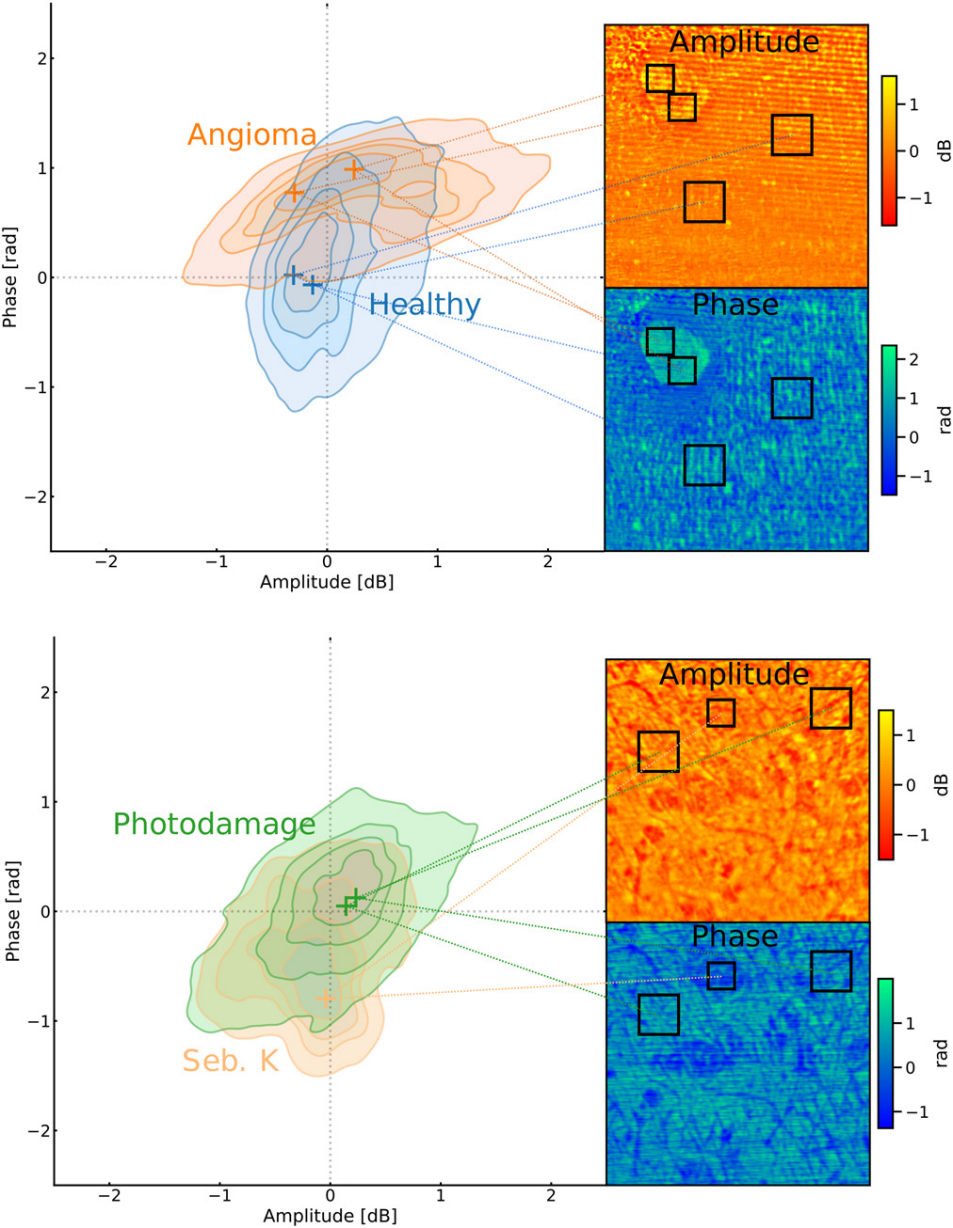
The distribution of skin pathologies vs surrounding healthy skin in the THz amplitude-phase map.

In order to statistically compare the THz properties of different lesions, multiple rectangular regions-of-interest (ROI) were manually selected from each lesion identified by the dermatologist (Fig. 7). All ROIs belonging to a single lesion type were pooled together and the corresponding amplitude-phase distribution was approximated with a Gaussian kernel density estimate (KDE, Fig. 8). The same KDE approximation was repeated for the images obtained from texture analysis.

## Data Availability

The data that support the findings of this study are available from the corresponding authors upon reasonable request.
